# Validity of the ESC Risk Assessment in Idiopathic Pulmonary Arterial Hypertension in China

**DOI:** 10.3389/fcvm.2021.745578

**Published:** 2021-11-22

**Authors:** Su-Gang Gong, Wen-Hui Wu, Chao Li, Qin-Hua Zhao, Rong Jiang, Ci-Jun Luo, Hong-Ling Qiu, Jin-Ming Liu, Lan Wang, Rui Zhang

**Affiliations:** ^1^Department of Pulmonary Circulation, Shanghai Pulmonary Hospital, Tongji University School of Medicine, Shanghai, China; ^2^Tongji University School of Medicine, Shanghai, China

**Keywords:** pulmonary arterial hypertension, idiopathic pulmonary arterial hypertension, risk assessment, guideline, prognosis

## Abstract

**Background:** The 2015 European pulmonary hypertension (PH) guidelines recommend a risk stratification strategy for pulmonary arterial hypertension (PAH). We aimed to investigate the validation and potential prognostic information in Chinese patients.

**Methods:** The risk assessment variables proposed by the PH guidelines were performed by using the WHO function class, 6-min walking distance, brain natriuretic peptide or its N-terminal fragment, right arterial pressure, cardiac index, mixed venous saturation, right atrium area, pericardial effusion, peak oxygen consumption, and ventilatory equivalents for carbon dioxide. An abbreviated version also was applied.

**Results:** A total of 392 patients with idiopathic PAH (IPAH) were enrolled between 2009 and 2018. After a median interval of 13 months, re-evaluation assessments were available for 386 subjects. The PAH guidelines risk tool may effectively discriminate three risk groups and mortality (*p* < 0.001) both at the baseline and re-evaluation. Meanwhile, its simplified risk version was valid for baseline and accurately predicted the risk of death in all the risk groups (*p* < 0.001). At the time of re-evaluation, the percentage of low-risk group has an increase, but a greater proportion achieved the high-risk group and a lesser proportion maintained in the intermediate-risk group.

**Conclusion:** The 2015 European PH guidelines and its simplified version risk stratification assessment present an effective discrimination of different risk groups and accurate mortality estimates in Chinese patients with IPAH. Changes of risk proportion at re-evaluation implicated that natural treatment decisions may not be consistently with goal-oriented treatment strategy.

## Introduction

The assessment of the prognosis of patients has been considered as an important section in patients with pulmonary arterial hypertension (PAH); different baseline and follow-up variables have been utilized individually or combined to predict outcome. Up to date, the 2015 European Society of Cardiology (ESC)/European Respiratory Society (ERS) pulmonary hypertension (PH) guidelines proceedings summarized risk stratification strategy advances (1), each focusing on different countries or registries, including the Registry to Evaluate Early and Long-Term Pulmonary Arterial Hypertension Disease Management (REVEAL) studies (2, 3), the Swedish PAH Registry (4), the Comparative, Prospective Registry of Newly Initiated Therapies for Pulmonary Hypertension (COMPERA) Registry (5), and the French PH Network (FPHN) (6). The updated analysis of risk stratification recommended a flexible and comprehensive approach by using the clinical features such as right ventricular function, hemodynamic parameters, biomarkers, and exercise. Based on the cutoff values gathered from the 2015 ESC/ERS guidelines, three risk categories were defined as a low-, intermediate-, or high-risk group (1).

The accuracy of this risk assessment strategy has been validated by the COMPERA Registry and mortality rate was significantly different between the three risk strata in baseline and follow-up (5). However, the COMPERA study used an abbreviated version risk analysis including six variables such as the WHO function class (FC), 6-min walk distance (6MWD), brain natriuretic peptide (BNP) or N-terminal proBNP (NT-proBNP), right arterial pressure (RAP), cardiac index (CI), and mixed venous oxygen saturation (S_V_O_2_), not capturing disease progression, syncope, echocardiography, and cardiopulmonary exercise testing (CPET) data. These findings confirm and extend previous study by Kylhammar et al. (4), who used the same subset of parameters [plus right atrial area and the presence/absence of pericardial effusion (PE)]. Although simplified variables could discriminate the risk groups, the most reliable dataset from echocardiography and CPET needed to determine (1). Patients with PAH usually show a typical pattern with low peak oxygen uptake [peak oxygen consumption (VO_2_)], providing prognostic information and therapeutic decision-making (1, 7). Echocardiography remained an important determinant as right ventricular function was key prognostic variables (8, 9). Similarly, the risk assessment of the French PH Network proposed by the European PH guidelines in patients with idiopathic PAH (IPAH) was available to work at baseline and follow-up (6). In fact, the risk stratification tool itself has a level of evidence C; also, the cutoff points are derived from several studies (1). However, it is unknown to validate the efficiency of this instrument in a real-world cohort in specific treatment era, especially in China.

The principle aim of this study was to apply the risk assessment from the 2015 ESC/ERS guidelines to a newly diagnosis cohort of patients with IPAH in China. We attempted to test the discrimination of the risk instrument presented in guidelines and to explore the potential prognostic changes at follow-up.

## Materials and Methods

### Study Patients

All the newly diagnosed patients with IPAH (≥18 years of age at diagnosis) were retrospectively reviewed in the Shanghai Pulmonary Hospital between January 2009 and September 2018. IPAH at baseline was set by right heart catheterization (RHC) according to standard criteria: a mean pulmonary artery pressure (mPAP) ≥25 mm Hg and pulmonary vascular resistance (PVR) >3 Wood units at rest in the presence of a normal pulmonary artery wedge pressure (PAWP) ≤ 15 mm Hg (1). Patients were excluded if they have definite causes for PAH such as connective tissue disease and congenital heart disease, those with portopulmonary hypertension, chronic pulmonary thromboembolism, and pulmonary hypertension due to left heart diseases and lung diseases and/or hypoxemia. Major endpoint was defined as all-cause mortality and no patients received lung or heart–lung transplantation. This study was conducted according to the principles of the Declaration of Helsinki and was approved by the Shanghai Pulmonary Hospital Ethics Committee (K19-054). Written informed consent was obtained from all the participants.

### Risk Stratification

Risk assessment was performed according to the 2015 ESC/ERS PH guidelines and patients were categorized as “low risk,” “intermediate risk,” or “high risk” in Table 1 (5). An abbreviated version of this guideline risk stratification strategy used the WHO FC, 6MWD, BNP or NT-proBNP, RAP, CI, and S_V_O_2_. The cutoff values proposed in the guidelines were graded as 1, 2, and 3 (1 = low risk, 2 = intermediate risk, and 3 = high risk). When the baseline 6-MWD did not detect, it was considered as a grade 3 (4). For each patient, the sum of all the grades was divided by the number of available variables. The mean grade was rounded to the next integer to define the risk group. For the follow-up risk stratification, we chose the visit that included follow-up hemodynamics after the baseline risk assessment at least 3 months. If no hemodynamic follow-up was available, we selected the follow-up visit that contained most of the data such as echocardiography or CPET. Variables listed in the guidelines that are not captured both at the baseline and follow-up are disease progression and syncope.

**Table 1:** Variables and cutoff values from the risk assessment from the ESC/ERS 2015 guidelines^*^.

	**Low risk**	**Intermediate risk**	**High risk**
WHO FC	I, II	III	IV
6MWD, meter	>440	165–440	<165
BNP, ng/L	<50	50–300	>300
NT-proBNP, ng/L	<300	300–1,400	>1,400
**Hemodynamics**			
RAP, mmHg	<8	8–14	>14
CI, L/min/m^2^	≥2.5	2.0–2.4	≤2.0
S_V_O_2_, %	>65	60–65	<60
**Imaging (echocardiography)**			
RA area, cm^2^	<18	18–26	>26
Pericardial effusion	No	No or minimal	Yes
**Cardio-pulmonary exercise testing**			
Peak VO_2_, mL/min/kg	>15 (>65% pred.)	11–15 (35–65% pred.)	<11 (<35% pred.)
VE/VCO_2_ slope	<36	36–44.9	≥45

* *Simplified version included the WHO FC, 6MWD, NT-proBNP, BNP, RAP, CI, and S_V_O_2_%*.

*BNP, brain natriuretic peptide; CI, cardiac index; ESC, European Society of Cardiology; ERS, European Respiratory Society; 6MWD, 6-minute walk distance; NT-proBNP, N-terminal pro-BNP; RA, right atrium; RAP, right atrial pressure; SvO_2_, mixed venous oxygen saturation; VE/VCO_2_, ventilatory equivalent for carbon dioxide; VO_2_, oxygen consumption; FC, function class*.

### Statistical Analysis

Continuous variables are expressed as mean ± SD or medians with corresponding 25th and 75th percentiles [interquartile range (IQR)]. Categorical variables are expressed as numbers and percentages. When data were not normally distributed, a non-parametric test was used. Changes between baseline and re-evaluation were assessed by using the chi-squared test where appropriate. Survival analyses were performed by using the Kaplan–Meier method, truncated at 5 years, and were compared by using the log-rank test. Survival time was calculated from the date of diagnostic RHC to the date of final follow-up and re-evaluation to final follow-up. Survival was compared for patients who were remained in the low-, intermediate-, or high-risk group, respectively, improved to the low- or intermediate-risk group, or worsened to the intermediate- or high-risk group. The univariate and multivariate Cox proportional hazards regression analysis was performed to assess the risk of death by using the respective low-risk group as reference. Patients were censored at termination December 31, 2018. A *p*-value < 0.05 was considered as statistically significant. All the analyses were performed by using the Statistical Package for the Social Sciences (SPSS) version 14.0 statistical software package (SPSS, Chicago, Illinois, USA).

## Results

### Study Patients

The baseline data reviewed total newly 392 patients with IPAH who fulfilled the criteria including 11 variables of interest for this study (Table 1). Out of the 10 variables at baseline, at least 4 variables were available in all the 392 patients, at least 10 in 59 (15%) patients and at least 8 in 177 (45%) patients. All the patients underwent the RHC examination. The characteristics of these patients in baseline were shown in Table 2. At the time of diagnosis, most patients (67%) were women and mean age was 40 years old. A total of 260 (67%) patients were in the WHO FC III or IV, whereas 34% patients were in the WHO FC I-II. All cause death survival for the overall study patients (*n* = 392) was shown in Supplementary Figure 1. For the initial treatment, 258 (66%) patients received monotherapy, 101 (26%) patients received combination therapy, and 33 (8%) patients received no specific/calcium channel blocker (CCB) therapy. At baseline, 186 (48%) patients used phospodiesterase 5 inhibitor (PDE5i) treatment in monotherapy group and 68 (17%) patients used PDE5i plus endothelin receptor antagonist (ERA) (Supplementary Table 1).

**Table 2:** Characteristics of patients with IPAH in baseline risk stratification.

	***N***	**Low risk**	**Intermediate risk**	**High risk**	**All**
Subjects, *n* (%)		96 (25)	267 (68)	29 (7)	392
Age, years		35 ± 14	42 ± 16	39 ± 16	40 ± 16
Female, *n* (%)		70 (73)	180 (67)	14 (48)	264(67)
BMI, kg/m^2^		23 ± 6	22 ± 4	23 ± 3	23 ± 4
**WHO FC**, ***n*** **(%)**					
Class I–II		69 (72)	63 (24)	0 (0)	132 (34)
Class III		27 (28)	188 (70)	19 (66)	234 (60)
Class IV		0 (0)	16 (6)	10 (35)	26 (7)
6MWD, meters	392	436 ± 97	364 ± 100	280 ± 95	379 ± 107
BNP, ng/L	164	46 (24, 94)	262 (149, 438)	661 (306, 880)	211 (65, 426)
NT-proBNP, ng/L	250	162 (40, 267)	1096 (542, 1892)	2428 (1949, 3771)	748 (255, 1679)
**Hemodynamics**					
RAP, mmHg	389	4 (2, 7)	6 (4, 10)	15 (12, 17)	6 (3, 10)
mPAP, mmHg	392	53 (45, 63)	58 (50, 69)	63 (56, 80)	58 (48, 68)
PAWP, mmHg	392	7 (6, 10)	8 (5, 10)	10 (7, 11)	8 (5, 10)
CI, L/min/m^2^	388	3.2 (2.8, 3.7)	2.2 (1.9, 2.7)	1.6 (1.5, 1.8)	2.4 (1.9, 3.0)
PVR, Wood units	392	9 (7, 12)	15 (11, 18)	20 (16, 26)	14 (9, 18)
S_V_O_2_, %	388	72 (68, 76)	60 (56, 65)	46 (42, 52)	62 (56, 69)
**Echocardiographic variables**					
RA area, cm^2^	235	16 (13, 20)	23 (18, 33)	38 (29, 47)	22 (16, 30)
No PE, *n* (%)		90 (94)	163 (61)	7 (24)	260 (66)
Minimal PE, *n* (%)		1 (1)	73 (27)	15 (52)	89 (25)
PE, *n* (%)		0 (0)	5 (2)	3 (10)	8 (2)
**Cardiopulmonary exercise testing**					
Peak VO_2_, mL/min/kg	100	18 ± 4	12 ± 3	9 ± 2	14 ± 4
VE/VCO_2_ slope	100	36 ± 7	62 ± 36	79 ± 22	56 ± 33
Initial therapies (within 3 months after diagnosis), *n* (%)
No specific/CCB therapy		13 (14)	20 (8)	0 (0)	33 (8)
Monotherapy		68 (71)	169 (63)	21 (72)	258 (66)
Combination therapy		15 (16)	78 (29)	8 (28)	101 (26)

*Values are expressed as mean ± SD, medians (interquartile range), or n (%), unless otherwise stated*.

*BMI, body mass index; BNP, brain natriuretic peptide; CCB, calcium channel blocker; CI, cardiac index; mPAP, mean pulmonary arterial pressure; 6MWD, 6-minute walk distance; NT-proBNP, N-terminal pro-BNP; PAWP, pulmonary artery wedge pressure; PE, pericardial effusion; PVR, pulmonary vascular resistance; RA, right atrium; RAP, right atrial pressure; SvO_2_, mixed venous oxygen saturation; VE/VCO_2_, ventilatory equivalents for carbon dioxide; VO_2_, oxygen consumption; FC, function class; IPAH, idiopathic pulmonary arterial hypertension*.

### Risk Assessment at Baseline and Mortality

**Figure 1:**
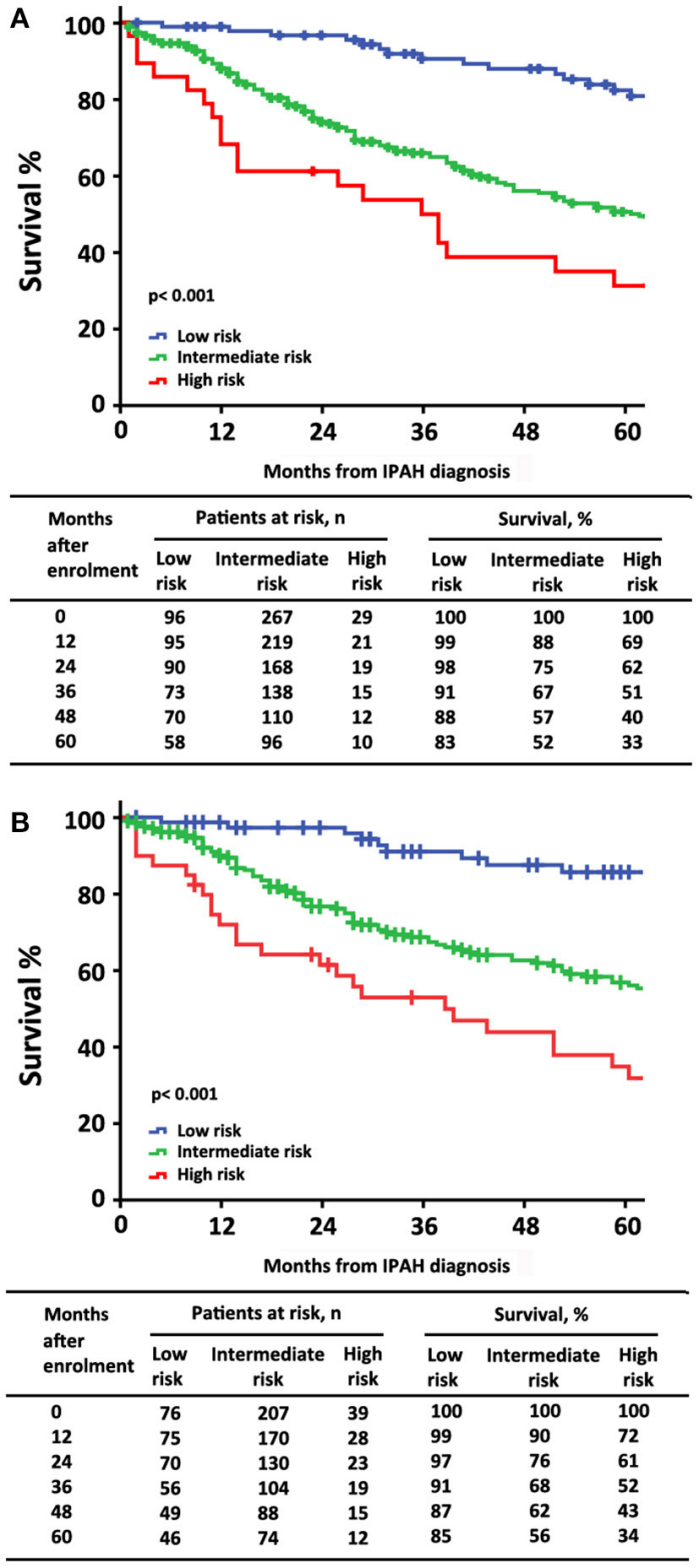
The survival estimates in patients with idiopathic pulmonary arterial hypertension at baseline according to **(A)** the 2015 European Society of Cardiology/European Respiratory Society (ESC/ERS) risk stratification strategy; **(B)** a simplified version.

At the time of diagnosis, 96 (25%) of patients were in the low-risk group, 267 (68%) of patients were in the intermediate-risk group, and 29 (7%) of patients were in the high-risk group, respectively (Table 2). After the diagnosis of IPAH within 5 years, 141 (36%) patients had died, 19 (20%) patients were in the low-risk group, 104 (39%) patients were in the intermediate-risk group, and 18 (62%) patients were in the high-risk group. In the low-risk group, the survival rate at 1-, 2-, 3-, 4-, and 5-year was 99, 98, 91, 88, and 83%, respectively. The corresponding survival was 88, 75, 67, 57, and 52% in the intermediate-risk group, respectively, and 69, 62, 51, 40, and 33% in the high-risk group, respectively (*p* < 0.001 for all the group comparisons). The predictive values of each variable at baseline are shown in Figure 1A.

**Figure 2:**
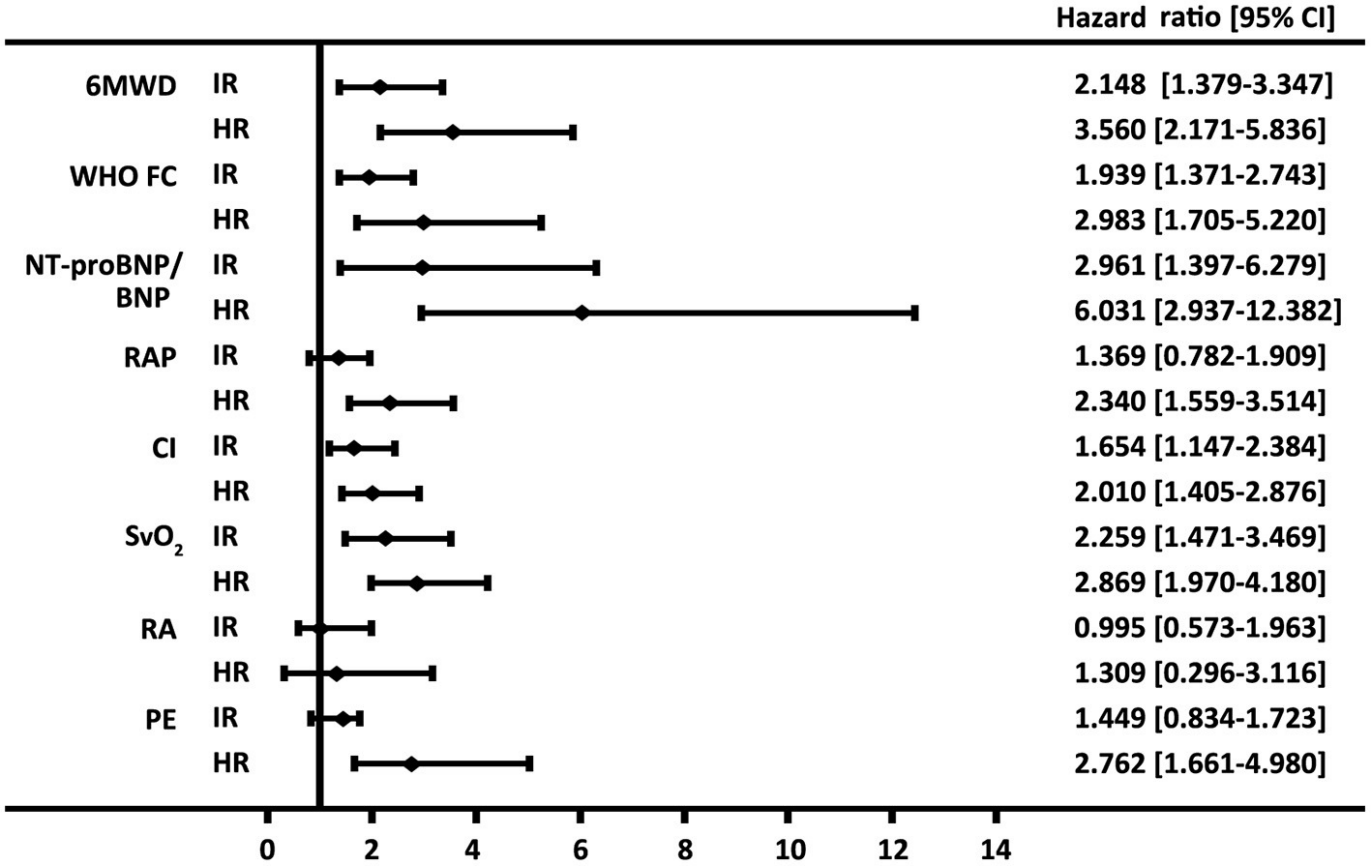
Forest plot based on the prognostic values of 6-min walk distance (6MWD), the WHO function class (FC), brain natriuretic peptide (BNP) or N-terminal proBNP (NT-proBNP), right arterial pressure (RAP), cardiac index (CI) and mixed venous oxygen saturation (S_V_O_2_), right atrium (RA) area, and pericardial effusion (PE) in the intermediate-risk (IR) and high-risk (HR) groups. Values for the variables were obtained from baseline. The reference value is from the respective low-risk group.

Similarly, by using simplified version, all the six variables were available in 322 patients, 76 (24%) of patients were in the low-risk group, 207 (64%) of patients were in the intermediate-risk group, and 39 (12%) of patients were in the high-risk group, respectively (Supplementary Table 2). Accordingly, the survival differences in the three risk categories were still statistical significant (*p* < 0.001 for all the group comparisons; Figure 1B). The predictive values of each variable in those patients at baseline are shown in Figure 2.

### Risk Assessment at Re-evaluation and Mortality

**Figure 3:**
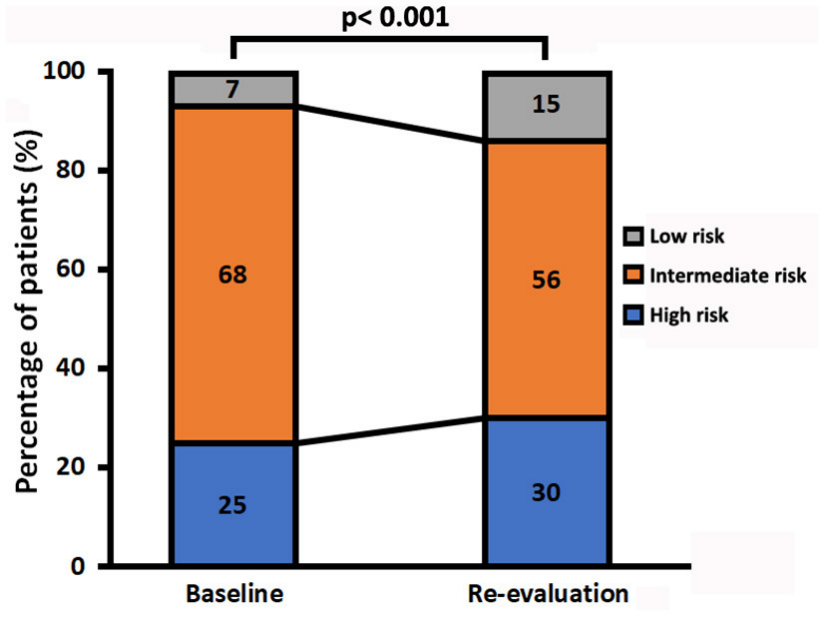
Change in the three risk groups in patients with idiopathic pulmonary arterial hypertension from baseline to re-evaluation.

At the end of follow-up, among the 106 patients with missing re-evaluation information, 24 (6%) patients died, 57 (15%) patients were below two variables, and 25 (6%) patients lost to follow-up for other specified reasons (Supplementary Figure 2). For re-evaluation assessment, out of the 10 variables, at least 2 variables were available in 286 patients, at least 8 variables were available in 11 (4%) patients, and at least 4 variables were available in 210 (73%) patients, respectively. Median interval between diagnosis and re-evaluation was 13 [5, 33] months. At the time of re-evaluation, 85 (30%) patients were in the low-risk group, 159 (56%) patients were in the intermediate-risk group, and 42 (15%) patients were in the high-risk group, respectively. There were increased 5% patients attaining high-risk group and decreased 12% patients with intermediate-risk group, although the percentage of low-risk group has an increased 8% (*p* < 0.001, Figure 3). Only 46 (16%) of these re-evaluation patients were available for hemodynamic data; however, 186 (65%) of these re-evaluation patients were available for right area, 253 (88%) of these re-evaluation patients were available for PE, and 88 (31) of these re-evaluation patients were available for CPET.

**Figure 4:**
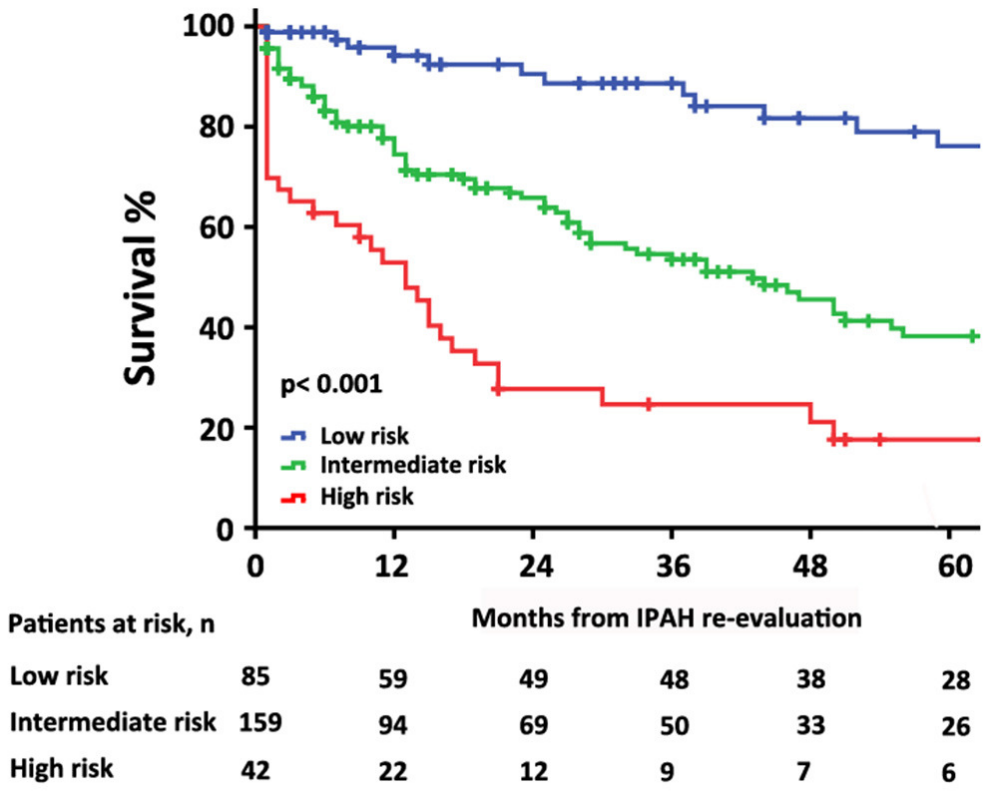
Survival estimates in patients with idiopathic pulmonary arterial hypertension at re-evaluation according to the 2015 ESC/ERS risk stratification strategy.

**Figure 5:**
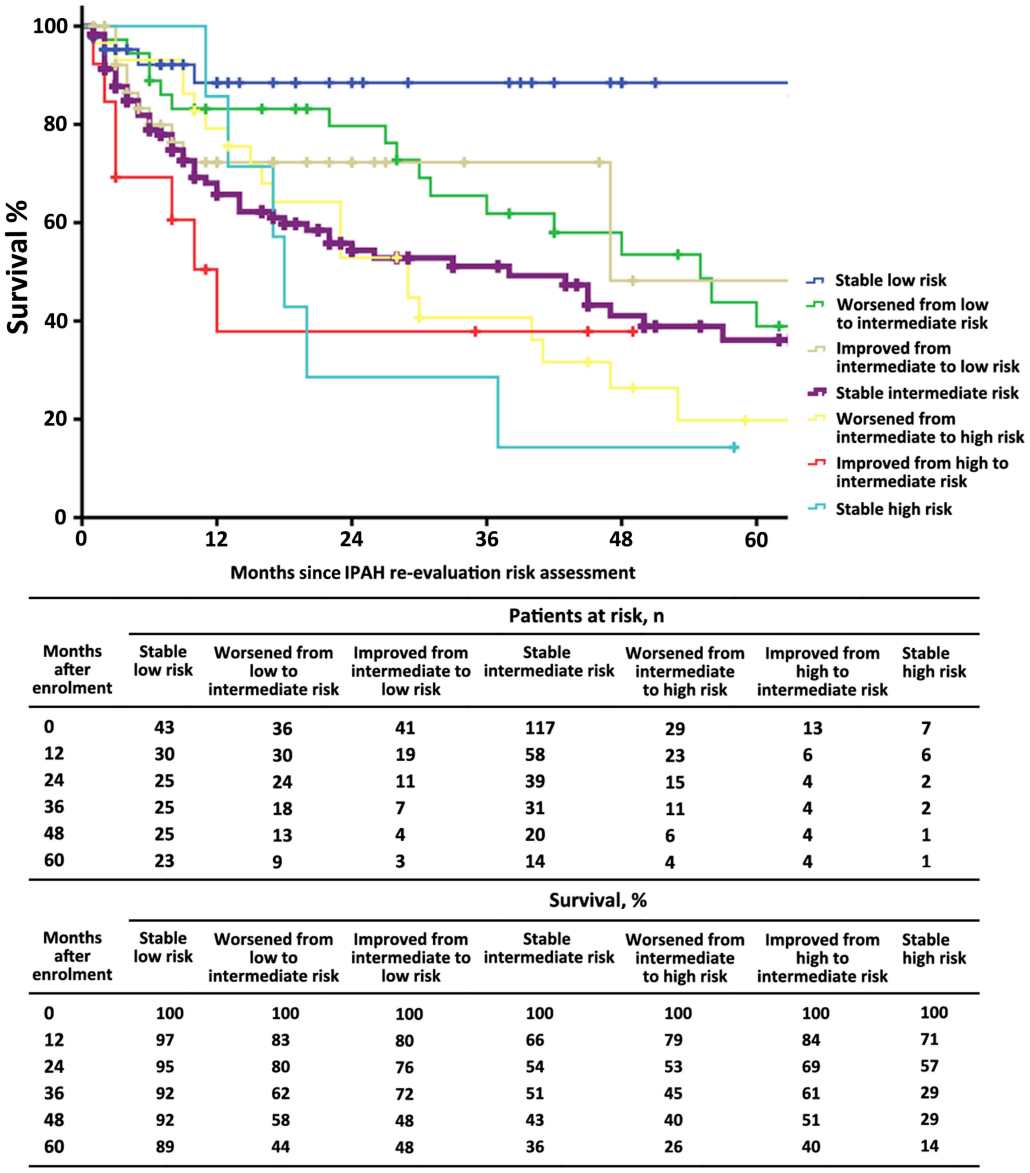
The survival estimates in patients with idiopathic pulmonary arterial hypertension according to the 2015 ESC/ERS risk category from baseline to re-evaluation. This figure was based on *n* = 286 patients.

The characteristics of these patients in re-evaluation were shown in Table 3. At re-evaluation, 147 (51%) patients received monotherapy, 126 (44%) patients received combination therapy, and 13 (4%) patients received no specific/CCB therapy. Within combination therapy group, 102 (36%) patients used PDE5i plus. Compared with those variables at baseline, there was significant improvement in 6MWD, CI, PVR, S_V_O_2_, and proportion of combination therapy (Supplementary Table 3). After re-evaluation of these patients within 5 years, 36 (13%) patients had died, 4 (8%) patients were in the low-risk group, 16 (10%) patients were in the intermediate-risk group, 7 (17%) patients were in the high-risk group, and 9 (3%) patients in censor. In the low-risk group, the survival rate at 1-, 2-, 3-, 4-, and 5-year was 94, 91, 89, 82, and 76%, respectively. The corresponding survival was 75, 66, 54, 46, and 38% in the intermediate-risk group, respectively, and 53, 28, 25, 21, and 18% in the high-risk group, respectively (*p* < 0.001 for all the group comparisons; Figure 4). The predictive values of each variable from the multivariate Cox proportional hazards regression analysis at re-evaluation are shown in Supplementary Figure 3. The WHO FC, 6MWD, NT-proBNP/BNP, and SVO_2_ were independent predictors. From baseline to re-evaluation, the changes in the risk assessment were associated with a shift in the mortality risk (*p* < 0.001 for all the group comparisons; Figure 5).

**Table 3:** Characteristics of patients with IPAH in re-evaluation risk stratification.

	***N***	**Low risk**	**Intermediate risk**	**High risk**	**All**
Subjects, *n* (%)		85 (30)	159 (56)	42 (15)	286
**WHO FC**, ***n*** **(%)**					
Class I–II		48 (56)	28 (18)	0 (0)	76 (27)
Class III		8 (9)	94 (59)	20 (48)	122 (43)
Class IV		0 (0)	6 (4)	19 (45)	25 (9)
6MWD, meters	135	472 ± 69	370 ± 106	204 ± 154	396 ± 120
BNP, ng/L	43	25 (13, 42)	297 (150, 453)	726 (385, 874)	184 (64, 453)
NT-proBNP, ng/L	224	74 (40, 142)	1,160 (541, 2,403)	1,691 (1,444, 2,844)	806 (146, 2,326)
**Hemodynamics**					
RAP, mmHg	46	5 (3, 7)	10 (6, 12)	12 (5, 14)	6 (4, 11)
mPAP, mmHg	46	43 (34, 52)	64 (57, 70)	78 (68, 84)	57 (40, 65)
PAWP, mmHg	46	9 (7, 11)	10 (6, 11)	10 (8, 14)	10 (7, 11)
CI, L/min/m^2^	46	3.5 (3.2, 4.6)	2.3 (1.9, 2.6)	1.9 (1.6, 2.0)	2.6 (2.2, 3.4)
PVR, Wood units	46	5 (4, 8)	15 (12, 20)	21 (17, 22)	10 (5, 15)
S_V_O_2_, %	46	74 (71, 78)	60 (52, 63)	50 (41, 58)	65 (58, 74)
**Echocardiographic variables**					
RA area, cm^2^	186	15 (12, 18)	22 (18, 29)	45 (37, 52)	23 (17, 34)
No PE, *n* (%)		74 (87)	91 (57)	19 (45)	170 (67)
Minimal PE, *n* (%)		2 (2)	45 (28)	12 (29)	73 (29)
PE, *n* (%)		0 (0)	3 (2)	7 (17)	10 (4)
**Cardiopulmonary exercise testing**					
Peak VO_2_, mL/min/kg	88	17 ± 3	12 ± 3	9 ± 1	13 ± 4
VE/VCO_2_ slope	88	35 ± 5	56 ± 25	81 ± 48	52 ± 28
Therapies (within 3 months after re-evaluation), *n* (%)					
No specific/CCB therapy		5 (6)	7 (4)	1 (2)	13 (4)
Monotherapy		54 (64)	77 (48)	16 (38)	147 (51)
Combination therapy		26 (31)	75 (47)	25 (60)	126 (44)

*Values are expressed as mean ± SD, medians (interquartile range), or n (%), unless otherwise stated*.

*BMI, body mass index; BNP, brain natriuretic peptide; CCB, calcium channel blocker; CI, cardiac index; mPAP, mean pulmonary arterial pressure; 6MWD, 6-minute walk distance; NT-proBNP: N-terminal pro-BNP; PAWP, pulmonary artery wedge pressure; PE, pericardial effusion; PVR, pulmonary vascular resistance; RA, right atrium; RAP, right atrial pressure; SvO_2_, mixed venous oxygen saturation; VE/VCO_2_, ventilatory equivalents for carbon dioxide; VO_2_, oxygen consumption; FC, function class*.

## Discussion

There was much evidence to support that the multiparametric approach stratified the patients with PAH in different risk groups for mortality. According to the risk status, different strategies can be utilized to guide therapeutic decisions (8). However, the validation of these comprehensive risk assessments for Chinese patients with IPAH is unclear. Among the above three registries, IPAH was a major etiology of PAH, such as 77% in the FPHN, 67% in the COMPERA Registry, and 51% in the Swedish PAH Registry, which indicated that IPAH was a special type and provided available strategy (4–6). The main findings of this study can be demonstrated as follows: (1) the 2015 European PH guidelines risk stratification effectively discriminated a low, intermediate, and high risk at baseline and re-evaluation assessments; (2) accurately predicted the risk of death in patients with IPAH; (3) its simplified version risk strategy was valid for baseline; and (4) the percentage of the low-risk group has an increase at re-evaluation, but a greater proportion of patients achieved the high-risk group and a lesser proportion maintained in the intermediate-risk group. Despite of the methodical risk assessments that are applicable for Chinese patients with IPAH, actual treatment seems not consistent with this goal-oriented treatment strategy.

A comprehensive assessment is used, since no single variable provides sufficient diagnostic and prognostic information. As we known, the 2015 ESC/ERS guidelines recommended 13 variables and the REVEAL risk score consisted of 19 variables (1, 2). The number of variables seems possible to discriminate risk groups accurately, but not all the variables may be done in PH centers. Except for the progression and syncope of the symptom, the variables selected in this study included all the RHC parameters, BNP or NT-proBNP, and 6WMD at baseline. Meanwhile, right atrium (RA) area was available in 235 (60%) patients, PE was available in 357 (91%) patients, and CPET was available in 100 (26%) patients (Tables 2, 3). It was significantly discrimination of different risk groups, i.e., 25% patients were in the low-risk group, 68% patients were in the intermediate-risk group, and 7% patients were in the high-risk group, respectively. If we used the simplified version of risk criteria, the proportion of the high-risk group was increased to 12% (Supplementary Table 2). Our results were closed to previous findings by the Swedish PH Registry, which the proportion of low-, intermediate-, and high-risk patients, respectively, was 23, 67, and 10% (530 patients with PAH and 49% patients with IPAH) (4). However, in the COMPERA IPAH subgroup study, the proportion of the high-risk group increased to 19% (5). The reasons for differences are partly attributed to different variables used for risk assessment or severity of different parameters. For example, 6MWD was 299 ± 123 m in the COMPERA IPAH subgroup, but 369 ± 107 m in this study. Our previous study has been reported that 6-min walk test values in Chinese patients with IPAH were significantly higher than those recorded in foreign registries (3, 10–12). Hence, it is necessary to discuss the feasibility of statistical risk calculation method. Given that echocardiography and CPET were not available for all the studies, most reliable indicators needed to further determine.

Regardless of whether regular follow-up, the three risk groups had significantly different long-term survival at baseline and in re-evaluation. It suggested that 13 variables of the 2015 ESC/ERS guidelines were relatively stable to discriminate risk stratification. However, we still found that an increased proportion of the high-risk group and a lesser proportion of patients with the intermediate-risk group, although the percentage of the low-risk group has an increase (Figure 3). The changes in risk category reflected that the patients in the low-risk group may be benefit from initial treatment, but those in the intermediate- and high-risk groups seemed not be sufficient. In this study, primary combinations of PAH-targeted drugs were observed in 26% of all the patients and in 28% of the high-risk patients, which implicated that combination treatment goal was not achieved in majority of our patients. Ample evidence was proposed to use of initial monotherapy or combination therapies in patients with naïve PAH (13–19). Initial combination therapy could improve exercise capacity and prognosis compared with initial monotherapy (14, 17). It is intelligible that the treatment goals are not always realistic and physicians may modify the therapeutic strategies with advanced disease or severe comorbidities. Certainly, the improved survival rates may be attributable to success of specific treatment and the increasing economic burden for patients cannot be ignored (10, 19). Even at the time of re-evaluation, over 50% patients are still in monotherapy or no specific therapies condition after all.

Of note, the parameters of echocardiography and CPET were used for risk stratification in this study including RA area, PE, peak VO_2_, and ventilatory equivalents for carbon dioxide (VE/VCO_2_) slope proposed by the 2015 PH guidelines. Presence of PE is common and thought to be an important indicator for right heart failure in patients with PAH (20, 21). Fenstad et al. reported that even modest degrees of pericardial fluid were associated with a significant increase in mortality in patients with PAH (22). In this study, we also found the degree of severe PE in the high-risk group that may be overestimated (Table 3, Figure 3). A preserved RA function is crucial to maintain sufficient right heart function, partly since the change of RA size alters the motion of the tricuspid annulus (23). Accordingly, impaired right ventricle systolic function and RA dilation (RA area > 18 cm^2^) were associated with worse long-term survival in patients with IPAH (24, 25). Grapsa et al. have reported that clinical deterioration was better associated with RA rather than RV remodeling in patients with PAH (26). There was no difference of RA area between baseline and re-evaluation (median was 22 cm^2^ at baseline and 23 cm^2^ in re-evaluation). Our data suggested that the parameters of PE and RA area were useful information for the risk stratification strategy.

Cardiopulmonary exercise testing may provide suggestive information in patients with PAH both at the circulation impairment and ventilatory inefficiency (27). Lower peak VO_2_ and higher VE/VCO_2_ slope were considered to establish the severity of exercise capacities or to assess outcomes (28–30). Wensel et al. have reported that average peak VO_2_ and VE/VCO_2_ slope during exercise were 11.2 ± 0.5 ml/min/kg and 54 ± 2 (2002) (31) and 13 ± 5 ml/min/kg and 54 ± 18 (2013) (30), respectively; also, patients with peak VO_2_ ≤ 10.4 ml/min/kg had poor survival. Our data showed that peak VO_2_ was 14 ± 4 ml/min/kg and VE/VCO_2_ slope was 54 ± 2 at baseline. Reference to the criteria of the 2015 ESC PH guidelines, the value of peak VO_2_ was arrived to the intermediate-risk group and VE/VCO_2_ slope was arrived to the high-risk group in this study. However, the two variables of CPET were all removed from the Cox multivariate model equation (at last step). This does not mean that CPET *per se* is not relevant, but that exercise function might not be superior to resting hemodynamics or echocardiography in this study. Although CPET is not widely utilized in patients with PAH, an increasing recognition of potential values should be emphasized (32). A total of 26% (100/392) patients of this study have CPET values, but further studies need still more valuable information to evaluate comprehensive score system for risk.

### Study Limitations

The major strengths of this study were the availability of complete data for invasive hemodynamics and non-invasive echocardiography and CPET variables at diagnosis in patients with IPAH. There are several limitations in this study. First, this is a retrospective study in a single center and the sample size was not large enough to provide sufficient numbers of the patients in three risk stratifications. Second, the follow-up assessments were not standardized and the proportion of RHC testing was lower at re-evaluation. However, we selected the follow-up visit that contained most of the data such as echocardiography or CPET. So, 88% patients at re-evaluation assessments had values of PE, 65% patients at re-evaluation assessments had values of RA area, and ~30% patients at re-evaluation assessments had values of CPET. Additionally, despite of median interval between diagnosis and re-evaluation was 13 months, ~15% of that was over 24 months, which may be biased toward the time-effect test. Finally, this study does not include prognostic variables, such as age, sex, comorbidities, disease progression, and syncope, and the individual risk is further modified by these factors. Further studies should organize more prospective studies or explore exiting registries in China.

## Conclusion

In conclusion, the present data show that the 2015 ESC/ERS PH guidelines and its simplified version risk stratification strategy may effectively discriminate different risk groups at baseline and re-evaluation. Meanwhile, this study validated an accurate prediction of mortality. Non-invasive echocardiography assessment might help to identify predictive usefulness of risk categorization strategies. The parameters of CPET seems to be less sensitive to the risk level designation, but need to be clarified in future and prospective studies. Changes of risk proportion at re-evaluation implicated that natural treatment decisions may not behave consistently with goal-oriented treatment strategy, but patients with IPAH may benefit from initial therapy.

## Data Availability Statement

The raw data supporting the conclusions of this article will be made available by the authors, without undue reservation.

## Ethics Statement

The studies involving human participants were reviewed and approved by K19-054. The patients/participants provided their written informed consent to participate in this study.

## Author Contributions

RZ and LW contributed to the study design, study conduct, supervision, scientific overview, data analysis, editing of the manuscript, and also directly involved in the recruitment and care of the patients. S-GG and W-HW contributed to enrolment of the patient, data analysis, scientific interpretation, drafting, and editing the original manuscript. CL, Q-HZ, RJ, C-JL, H-LQ, and J-ML contributed to recruitment of participants, data collection, curation, and formal analysis. All authors have reviewed the manuscript, approved the final version for submission, participated in the design of this study, patient enrolment, and meet criteria for authorship.

## Funding

This study was supported in part by the National Natural Science Foundation of China (82000059) (LW), the Project of International Cooperation (19410741000) (RZ) and (201409004100) (S-GG) in Science and Technology Commission Shanghai Municipality, and the Youth Scholar Program of Shanghai Pulmonary Hospital (fkgg1804) (RZ).

## Conflict of Interest

The authors declare that the research was conducted in the absence of any commercial or financial relationships that could be construed as a potential conflict of interest.

## Publisher's Note

All claims expressed in this article are solely those of the authors and do not necessarily represent those of their affiliated organizations, or those of the publisher, the editors and the reviewers. Any product that may be evaluated in this article, or claim that may be made by its manufacturer, is not guaranteed or endorsed by the publisher.
