# Supplementary material for: Validity of the ESC Risk Assessment in Idiopathic Pulmonary Arterial Hypertension in China
Source: Front Cardiovasc Med. 2021 Nov 22;8:745578. doi: 10.3389/fcvm.2021.745578 (PMC8645595; doi:10.3389/fcvm.2021.745578)
Supplement: Supplementary file 1 [file Data_Sheet_1.docx]

**Supplemental Material**

**Validity of ESC risk assessment in idiopathic pulmonary arterial hypertension in China**

Su-Gang Gong^1,#^, Wen-Hui Wu^1,#^, Chao Li^2^, Qin-Hua Zhao^1^, Rong Jiang^1^, Ci-Jun Luo^1^, Hong-Ling Qiu^1^, Jin-Ming Liu, Lan Wang^1,*^ Rui Zhang^1,*^

^1^Department of Pulmonary Circulation, Shanghai Pulmonary Hospital, Tongji University School of Medicine, Shanghai, 200433, China

^2^Tongji University School of Medicine, Shanghai, 200092, China

**^#^Drs Su-Gang Gong and Wen-Hui Wu contributed equally to this article.**

***Co-correspondence:**

Dr. Rui Zhang, MD, E-mail: [zhangrui@tongji.edu.cn](mailto:zhangrui@tongji.edu.cn);

and Dr. Lan Wang, E-mail: [wanglan198212@163.com](mailto:wanglan198212@163.com);

Department of Pulmonary Circulation, Shanghai Pulmonary Hospital, Tongji University School of Medicine, No. 507 Zhengmin Road, Shanghai, 200433, China; Telephone: +86-21-65115006, Fax: +86-21-65115018;


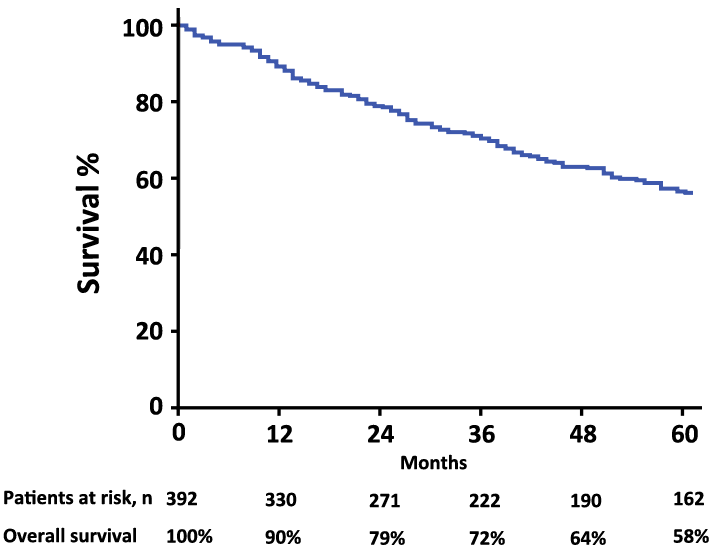


**Supplementary Figure S1.** Kaplan-Meier survival estimates of all patients with IPAH at baseline.


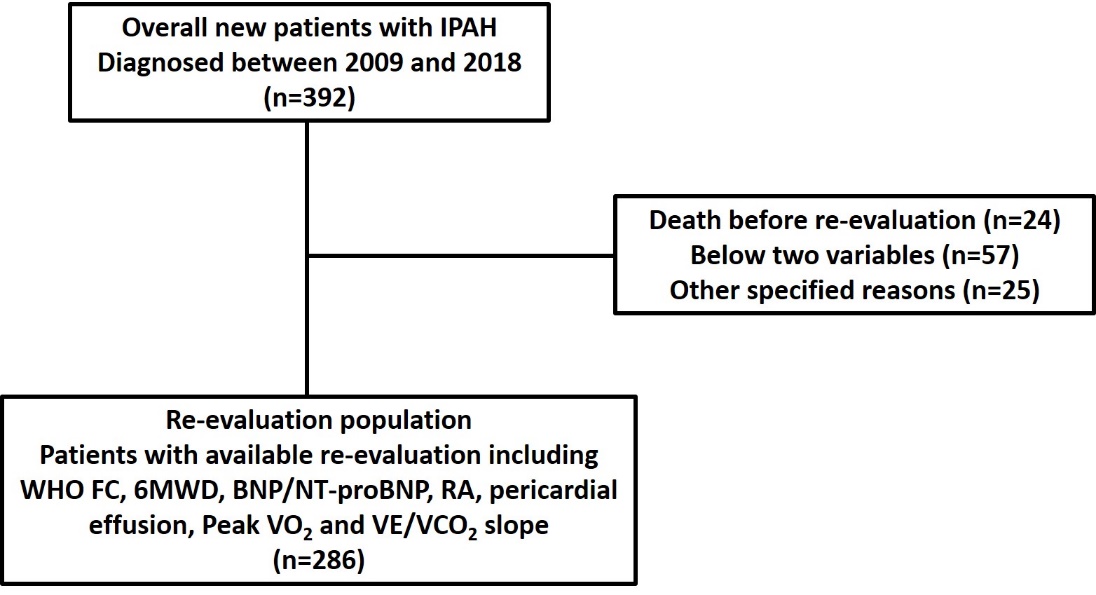


**Supplementary Figure S2.** Patient selection flow chart.

BNP: brain natriuretic peptide; IPAH: idiopathic pulmonary hypertension; 6MWD: 6-minute walking distance; NT-proBNP: N-terminal fragmental of pro-brain natriuretic peptide; RA: right atrium; VE/VCO_2_: ventilatory equivalents for carbon dioxide; VO_2_: oxygen consumption; WHO FC: World Health Organization functional class.


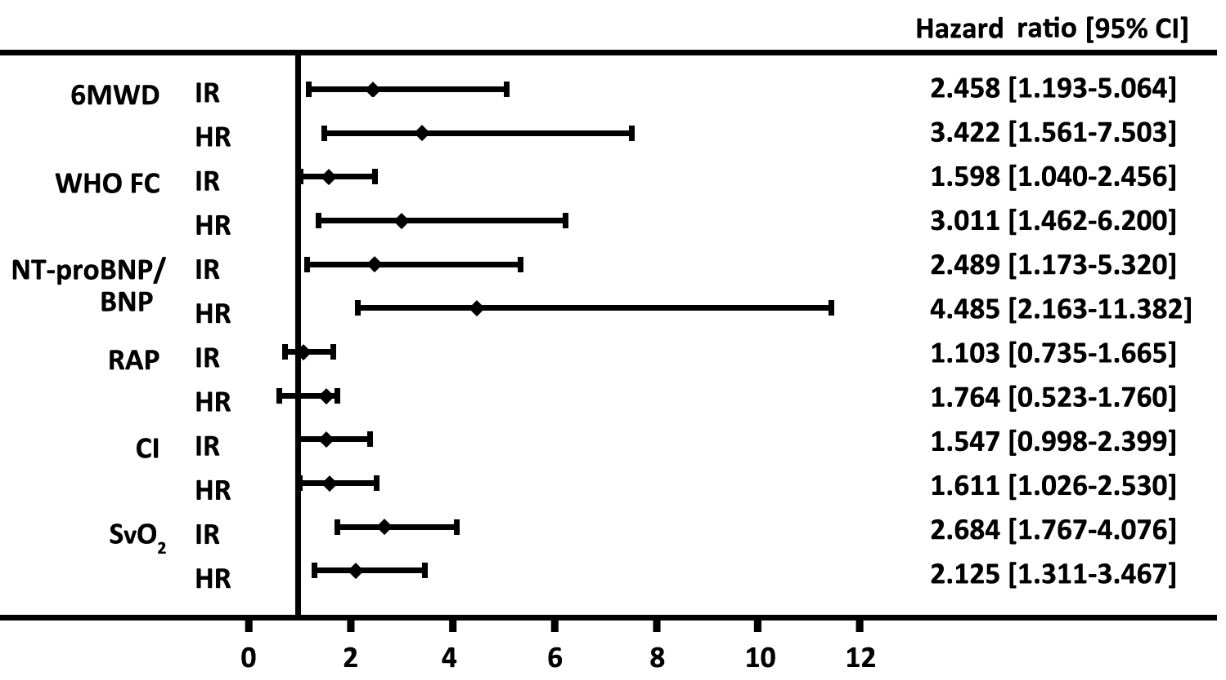


**Supplementary Figure S3.** Forest plot based on the prognostic values of 6-min walking distance (6MWD), World Health Organization (WHO) function class (FC), brain natriuretic peptide (BNP) or N-terminal fragment of proBNP (NT-proBNP), right arterial pressure (RAP), cardiac index (CI) and mixed venous oxygen saturation (S_V_O_2_) in the Intermediate risk (IR) and High risk (HR) groups. Values for the variables were obtained from re-evaluation. The reference value is from the respective Low risk group.

| **Supplementary Table S1. PAH-targeted treatment initiated within 3 months after PAH diagnosis and re-evaluation** | | |
| --- | --- | --- |
| **Treatment** | **Baseline**  **(392)** | **Re-evaluation (286)** |
| **Monotherapy, n (%)** |  |  |
| ERA^*^ | 56 (14) | 38 (13) |
| PDE5-I^#^ | 186 (48) | 107 (37) |
| Prostanoid^¶^ | 16 (4) | 10 (3) |
| **Dual combination, n (%)** |  |  |
| ERA+PDE5-I | 68 (17) | 102 (36) |
| PDE5-I+prostanoid | 2 (1) | 16 (6) |
| ERA+prostanoid | 31 (8) | 3 (1) |
| **Triple combination, n (%)** | 0 (0) | 5 (2) |
| **Calcium channel blockers, n (%)** | 5 (1) | 2 (1) |
| **No treatment, n (%)** | 28 (7) | 3 (1) |

ERA: endothelin-receptor antagonist; PDE5-I: phosphodiesterase type-5 inhibitor

^*^ERA treatment included 12 patients in PATENT Trial.

^#^PDE5-I group included 10 patients in SERAPHIN Trial.

^¶^Prostanoid treatment included 11 patients in UT Trial and 3 patients in GRIPHON Trial.

| Supplementary Table S2. Characteristics of IPAH patients in baseline risk assessment according to simplified version of 2015 ESC/ERS risk stratification | | | | |
| --- | --- | --- | --- | --- |
|  | **Low risk** | **Intermediate risk** | **High risk** | **All** |
| Subjects, n (%) | 76 (24) | 207 (64) | 39 (12) | 322 |
| Age, years | 36±15 | 42±16 | 39±14 | 38±14 |
| Female, n (%) | 53 (70) | 143 (69) | 19 (49) | 215 (67) |
| WHO FC, n (%) |  |  |  |  |
| Class I-II | 55 (72) | 50 (24) | 0 (0) | 105 (33) |
| Class III | 21 (28) | 147 (71) | 31 (80) | 199 (60) |
| Class IV | 0 (0) | 10 (5) | 8 (21) | 18 (6) |
| 6MWD, meters | 421±104 | 363±94 | 257±100 | 369±107 |
| BNP, ng/L | 46 (23, 81) | 204 (132, 412) | 661 (370, 844) | 160 (71, 390) |
| NT-proBNP, ng/L | 171 (51, 287) | 1154 (560, 1918) | 2309 (2095, 2703) | 1679 (264, 1893) |
| Hemodynamics | | | | |
| RAP, mmHg | 5 (3, 6) | 8 (4, 12) | 15 (10, 22) | 7 (4, 11) |
| mPAP, mmHg | 62 (44, 65) | 62 (52, 70) | 61 (58, 81) | 62 (51, 78) |
| PAWP, mmHg | 7 (5, 10) | 7 (5, 10) | 9 (7, 11) | 9 (6, 11) |
| CI, L/min/m^2^ | 3.1 (2.7, 3.7) | 2.2 (2.0, 2.4) | 1.8 (1.6, 1.9) | 2.4 (2.0, 3.1) |
| PVR, Wood units | 10 (7, 12) | 15 (12, 18) | 19 (15, 26) | 14 (10, 17) |
| S_V_O_2_, % | 71 (66, 77) | 61 (57, 67) | 47 (44, 55) | 64 (57, 70) |
| Initial therapies (within 3 months after diagnosis), n (%) | | | | |
| No specific therapy | 5 (7) | 17 (8) | 1 (3) | 23 (7) |
| CCB therapy | 5 (7) | 0 (0) | 0 (0) | 5 (16) |
| Monotherapy | 55 (72) | 120 (58) | 26 (67) | 201 (62) |
| Combination therapy | 11 (15) | 70 (34) | 12 (31) | 93 (29) |

Values are expressed as mean±SD, medians (interquartile range) or n (%), unless otherwise stated. BMI: body mass index; BNP: brain natriuretic peptide; CCB: calcium channel blocker; CI: cardiac index; mPAP: mean pulmonary arterial pressure; 6MWD: 6-minute walking distance; NT-proBNP: N-terminal fragmental of pro-brain natriuretic peptide; PAWP: pulmonary artery wedge pressure; PE: pericardial effusion; PVR: pulmonary vascular resistance; RAP: right atrial pressure; SvO_2_: mixed venous oxygen saturation; WHO FC: World Health Organization functional class.

| **Supplementary Table S3. Change in variable between baseline and re-evaluation** | | | |
| --- | --- | --- | --- |
|  | **Baseline** | **Re-evaluation** | **p-value** |
| Subjects, n | 392 | 286 |  |
| **Risk assessment, n (%)** | | | <0.001 |
| Low risk | 96 (25) | 85 (30) |  |
| Intermediate risk | 267 (68) | 159 (56) |  |
| High risk | 29 (7) | 42 (15) |  |
| **WHO FC, n (%)** | | | <0.001 |
| Class I-II | 132 (34) | 76 (27) |  |
| Class III | 234 (60) | 122 (43) |  |
| Class IV | 26 (7) | 25 (9) |  |
| 6MWD, meter | 379±107 | 396±120 | 0.009 |
| BNP, ng/L | 211 (65, 426) | 184 (64, 453) | 0.698 |
| NT-proBNP, ng/L | 748 (255, 1679) | 806 (146, 2326) | 0.098 |
| **Haemodynamics^*^** | | | |
| RAP, mmHg | 6 (3, 10) | 6 (4, 11) | 0.073 |
| mPAP, mmHg | 58 (48, 68) | 57 (40, 65) | 0.068 |
| PAWP, mmHg | 8 (6, 10) | 10 (7, 11) | 0.069 |
| CI, L/min/m^2^ | 2.4 (1.9, 3.0) | 2.6 (2.2, 3.4) | 0.009 |
| PVR, Wood units | 14 (9, 18) | 10 (5, 15) | 0.004 |
| S_V_O_2_, % | 62 (56, 69) | 65 (58, 74) | 0.038 |
| **Imaging (echocardiography)** | | | |
| RA area, cm^2^ | 22 (16, 30) | 23 (17, 34) | 0.224 |
| Pericardial effusion |  |  | <0.001 |
| No PE, n (%) | 260 (66) | 170 (67) |  |
| Minimal PE, n (%) | 89 (25) | 73 (29) |  |
| PE, n (%) | 8 (2) | 10 (4) |  |
| **Cardio-pulmonary exercise testing** | | | |
| Peak VO_2_, mL/min/kg | 14±4 | 13±4 | 0.668 |
| VE/VCO_2_ slope | 56±33 | 52±28 | 0.108 |
| **Therapies, n (%)** | | | <0.001 |
| No specific/CCB therapy | 33 (8) | 5 (2) |  |
| Monotherapy | 258 (66) | 155 (54) |  |
| Combination therapy | 101 (26) | 126 (44) |  |

^*^Haemodynamics of re-evaluation could be successfully measured in 44 patients.

Values are expressed as mean±SD, medians (interquartile range) or n (%), unless otherwise stated. BNP: brain natriuretic peptide; CCB: calcium channel blocker; CI: cardiac index; mPAP: mean pulmonary arterial pressure; 6MWD: 6-minute walking distance; NT-proBNP: N-terminal fragmental of pro-brain natriuretic peptide; PAWP: pulmonary artery wedge pressure; PE: pericardial effusion; PVR: pulmonary vascular resistance; RA: right atrium; RAP: right atrial pressure; SvO2: mixed venous oxygen saturation; VE/VCO2: ventilatory equivalents for carbon dioxide; VO2: oxygen consumption; WHO FC: World Health Organization functional class.
